# Ecological processes shaping highly connected bacterial communities along strong environmental gradients

**DOI:** 10.1093/femsec/fiae146

**Published:** 2024-10-30

**Authors:** Wenxue Wu, Chih-hao Hsieh, Ramiro Logares, Jay T Lennon, Hongbin Liu

**Affiliations:** State Key Laboratory of Marine Resource Utilization in South China Sea, Hainan University, Haikou 570228, Chinese mainland; Southern Marine Science and Engineering Guangdong Laboratory, Zhuhai 519082, Chinese mainland; School of Marine Science, Sun Yat-sen University, Zhuhai 519082, Chinese mainland; Institute of Oceanography, National Taiwan University, Taipei 106319, Taiwan; Institute of Marine Sciences, CSIC, Barcelona 08003, Spain; Department of Biology, Indiana University, Bloomington, IN 47405, United States; Southern Marine Science and Engineering Guangdong Laboratory, Zhuhai 519082, Chinese mainland; Department of Ocean Science, The Hong Kong University of Science and Technology, Kowloon 999077, Hong Kong

**Keywords:** metacommunity, heterogeneous selection, homogenizing dispersal, metabolic activity, river–sea continuum

## Abstract

Along the river–sea continuum, microorganisms are directionally dispersed by water flow while being exposed to strong environmental gradients. To compare the two assembly mechanisms that may strongly and differently influence metacommunity dynamics, namely homogenizing dispersal and heterogeneous selection, we characterized the total (16S rRNA gene) and putatively active (16S rRNA transcript) bacterial communities in the Pearl River–South China Sea Continuum, during the wet (summer) and dry (winter) seasons using high-throughput sequencing. Moreover, well-defined sampling was conducted by including freshwater, oligohaline, mesohaline, polyhaline, and marine habitats. We found that heterogeneous selection exceeded homogenizing dispersal in both the total and active fractions of bacterial communities in two seasons. However, homogeneous selection was prevalent (the dominant except in active bacterial communities during summer), which was primarily due to the bacterial communities’ tremendous diversity (associated with high rarity) and our specific sampling design. In either summer or winter seasons, homogeneous and heterogeneous selection showed higher relative importance in total and active communities, respectively, implying that the active bacteria were more responsive to environmental gradients than were the total bacteria. In summary, our findings provide insight into the assembly of bacterial communities in natural ecosystems with high spatial connectivity and environmental heterogeneity.

## Teaser

This study makes inferences about how highly connected bacterial communities are assembled along strong environmental gradients.

## Introduction

Microorganisms exhibit diverse and complex community structures across aquatic ecosystems. Although microorganisms can now be rapidly and accurately identified using high-throughput sequencing, ecological processes, which refer to the assembly of microbial communities remain complicated, especially in flowing environments where individuals are strongly influenced by currents and physical forces (Read et al. 2015, Savio et al. 2015, Henson et al. 2018, Liu et al. 2018, Gweon et al. 2021). Such conditions are common in rivers that are commonly characterized by strong environmental gradients. Therefore, the microbial communities in rivers are ideal models for quantitative metacommunity studies focusing on a set of local communities linked by dispersing individuals (Leibold et al. 2004, Vellend 2010). The metacommunity framework allows for tests of the relative importance of dispersal and selection on assembly processes (Stegen et al. 2013). Specifically, when movement is restricted, dispersal limitation leads to compositional variation among sites (Langenheder and Lindström 2019). When movement is relatively unrestricted, high rates of dispersal can homogenize compositions among sites (Heino et al. 2015). Conversely, ecological selection occurs when there are fitness differences between organisms that either constrain (i.e. homogeneous selection) or promote (i.e. heterogeneous selection) turnover in composition among sites with respect to phylogenetic relatedness of taxa (Zhou and Ning 2017).

It is particularly challenging to understand the ecological processes that underpin the bacterial communities involved in complex biogeochemical processes at river mouths (Raymond and Bauer 2001). Many studies have shown that bacterial communities in river–sea continua change dramatically across space (Alonso et al. 2010, Jeffries et al. 2016, Aguirre et al. 2017, Sia et al. 2019). Dispersal- and selection-inferred processes may contribute inversely to bacterial community variations in river–sea continua (Stadler and del Giorgio 2022). In detail, a river–sea continuum can be defined as a reach that characteristically ranges from freshwater to seawater. Transitions typically occur within a 100–200 km distance (Wu and Liu 2018), which is a relatively small geographical scale given that bacteria can easily disperse across such highly connected ecosystems (Heino et al. 2015). As a result of high rates of immigration and emigration, dispersal may homogenize the bacterial communities (Niño-García et al. 2016, Shen et al. 2018, Fodelianakis et al. 2019). However, river–sea continua are also characterized with strong environmental gradients involving variables like salinity (Langenheder et al. 2003, Paver et al. 2018), which can efficiently differentiate bacterial communities harboring taxa with distinct niches (Lozupone and Knight 2007, Thompson et al. 2017). Heterogeneous selection can emerge as a result of promoted turnover among bacteria with distantly phylogenetic relatedness, and may overpower the role of homogenizing dispersal. Although river–sea continua are an important link between terrestrial, freshwater, and marine ecosystems (Zutic and Legovic 1987, Telesh and Khlebovich 2010, Xenopoulos et al. 2017), the interaction of the underlying mechanisms that shape bacterial communities remains unclear.

The relative importance of certain ecological processes can differ based on the metabolic activity of microbial taxa (Muscarella et al. 2016, Wisnoski et al. 2020). To gain a comprehensive understanding and account for metabolic states, total and active bacterial communities are increasingly being studied together (Jones and Lennon 2010, Campbell et al. 2011, Lennon and Jones 2011, Campbell and Kirchman 2013, Richa et al. 2017, Locey et al. 2020). In some aquatic ecosystems, only a small proportion of the total bacterial community is potentially active, ranging from <5% (e.g. in oligotrophic open oceans) to >50% (e.g. in highly productive estuaries) (del Giorgio and Scarborough 1995, Smith and del Giorgio 2003). As a result, the total and active bacterial communities exhibit distinct structures. For example, the total community may contain slow-growing, dormant, and dead bacteria that can be transported across strong environmental gradients (Lennon et al. 2018, Nagler et al. 2018). Their reduced metabolic state may improve colonization success and dampen spatial variability of community structure (Carini et al. 2016), thereby reducing the expected signature of heterogeneous selection. Conversely, active communities exhibit faster and greater responses to changing environmental conditions (Hoshino and Matsumoto 2007, Franzosa et al. 2015, De Vrieze et al. 2016), and homogeneous selection that constrains community structure might be negated. Although many studies have described total and active bacterial communities concurrently, the differences in their assembly mechanisms were only compared in a small portion of previous studies (Logue and Lindström 2010, Jia et al. 2020, Locey et al. 2020, Stadler and del Giorgio 2022).

Here, we study the bacterial communities in the Pearl River–South China Sea Continuum (PSC). According to the Venice System for the classification of marine waters (Anonymous 1958), the PSC covers diverse habitats spanning a range of salinities, including freshwater (<0.5), oligohaline (0.5–5), mesohaline (5–18), polyhaline (18–30), and marine (>30), for approximate 120 km. Moreover, the PSC is strongly influenced by seasonal river runoff events (Sun et al. 2014), which may affect the balance between dispersal- and selection-inferred processes (Huber et al. 2020). We conducted surveys along the PSC during both summer (wet season) and winter (dry season). At each sampling station, we collected surface and bottom samples and characterized bacterial communities using high-throughput sequencing of the 16S rRNA genes (DNA) and transcripts (RNA). Despite several debatable limitations and caveats (Blazewicz et al. 2013), it is generally accepted that DNA-based data represent the total bacterial communities (including living, dormant, and dead organisms), whereas RNA-based data primarily account for putatively active bacteria (Kirchman 2016). We quantified the relative importance of five ecological processes: heterogeneous selection, dispersal limitation, undominated fraction, homogenizing dispersal, and homogeneous selection, using a two-step framework (Stegen et al. 2013, Stegen et al. 2016). We compared homogenizing dispersal versus heterogeneous selection in each of the four sets of bacterial communities (i.e. total and active bacteria in summer and winter seasons), and tested how homogeneous and heterogeneous selection (in each season) varied in total versus active communities. This study provides novel insights into the assembly of highly connected bacterial communities along strong environmental gradients.

## Materials and methods

### Field sampling of bacterial communities

**Figure 1.**
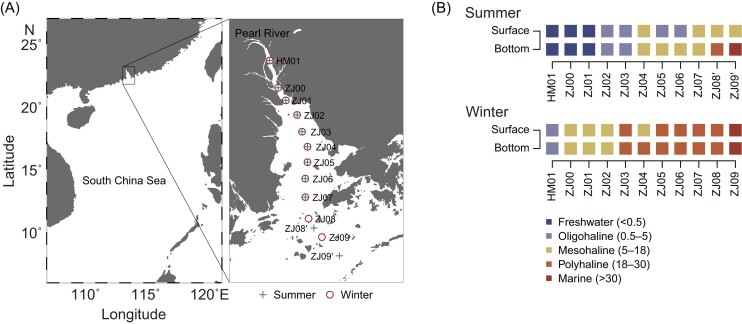
(A) Map showing the sampling locations in the Pearl River–South China Sea Continuum during the summer (indicated by blue crosses) and winter (indicated by red circles) seasons, and (B) the type of habitats (classified according to salinity shown in brackets) that each sampling location belongs to. Nine of the 11 stations sampled in each season overlap, while the remaining two are differently located. The map was generated using the Ocean Data View software (http://odv.awi.de).

Water samples for molecular analyses were taken from the PSC in 2014 during both the wet summer (i.e. between May 13 and 15) and dry winter (between December 29 and 31) (Fig. 1A). In each season, a total of 22 samples were collected from the surface (at 0.5 m depth) and bottom (5–21 m in the summer and 4.5–14 m in the winter; i.e. at 0.5–2 m above the sediment) layers across a transect (11 samples per layer). During summer and winter, 9 of the 11 stations were repeatedly surveyed, while the other two stations had a shift in geographical locations between two seasons. Considering context dependency in metacommunity studies (Heino et al. 2012), our PSC sampling design covered sequential freshwater, oligohaline, mesohaline, polyhaline, and marine habitats (according to salinity therein). After pre-filtering 100–800 ml water through an 80-µm mesh, bacterial cells were collected using 0.2-µm polycarbonate membranes (Millipore, Carrigtwohill, Cork, Ireland). After immersing the filters in RNA *later* solution (Ambion, Austin, TX, USA), they were immediately frozen in liquid nitrogen before being stored at −80°C until molecular analyses.

### Environmental distance calculation

To determine environmental contexts across the transect, we included a series of abiotic and biotic environmental variables, including temperature, salinity, chlorophyll *a*, NH_4_, NO_2_, NO_3_, PO_4_, suspended particulate matter, particulate organic carbon, particulate nitrogen, and cell numbers of heterotrophic bacteria, *Synechococcus*, and pigmented picoeukaryotes, which were analyzed as described previously (Wu and Liu 2018). Pearson’s correlations between environmental variables were analyzed and visualized via heatmaps.

To determine environmental distances between sampling sites, the values of each environmental variable were standardized with a mean of 0 and variance of 1, and the environmental distances were then calculated as the Euclidean distance between sites. This transformation enabled the linking of ecological processes (estimated as below) with environmental contexts (and hence, to a holistic environmental perspective) rather than to a single variable. The relationship between environmental and geographical distances was evaluated using Mantel test.

### Library construction and sequence processing

DNA/RNA extraction and cDNA synthesis procedures have been previously described in detail (Wu and Liu 2018). Barcoded primers 515F (5′-GTGCCAGCMGCCGCGGTAA-3′) and 806R (5′-GGACTACHVGGGTWTCTAAT-3′) (Caporaso et al. 2011) were used to amplify the V4 region of the 16S rRNA (gene), based on a dual-index strategy. Polymerase chain reaction (PCR) mixtures of 20 µl contained 1× PCR buffer, 1.5 mM MgCl_2_, 0.2 mM dNTP mix, 0.5 µM each of primer, 1 U Invitrogen Platinum *Taq* DNA polymerase (Life Technologies, Carlsbad, CA, USA), and DNA/cDNA templates (∼1–10 ng). PCR was performed as follows: 94°C for 3 min, followed by 30 cycles at 94°C for 45 s, 50°C for 60 s, and 72°C for 90 s, with a final extension step at 72°C for 10 min. For each sample, triplicate PCR products were pooled and sequenced on a HiSeq 2500 platform (Illumina, San Diego, CA, USA), generating 2 × 250 bp paired-end reads.

The Quantitative Insights Into Microbial Ecology (QIIME v. 1.9.1) pipeline was used to process the sequences (Caporaso et al. 2010). Using the default parameters, overlapping paired-end reads were merged using SeqPrep (https://github.com/jstjohn/SeqPrep). The merged sequences were then filtered using the following criteria: 1) Phred scores < 25; 2) homopolymers > 6; 3) with any mismatch in the primers; 4) with any error in the barcodes; 5) containing ambiguous bases; 6) too short (<270 bp) or too long (>320 bp). The filtered sequences were grouped into operational taxonomic units (OTUs) using Sumaclust (Mercier et al. 2013) at 99% similarity (Koeppel and Wu 2014, Louca et al. 2016). After removing singletons and doubletons, ChimeraSlayer was used to identify and remove chimeras based on representative sequences (i.e. the most abundant sequence in each OTU) (Haas et al. 2011). These representative sequences were annotated against the SILVA 119 database (Quast et al. 2013) using BLAST (*E* value = 10^−6^), and those affiliated with archaea, eukaryotes, chloroplasts, and mitochondria were removed from the OTU table. The remaining representative sequences were aligned with MAFFT 7 (Katoh and Standley 2013) using the FFT-large-NS-2 method. After the removal of columns that showed gaps in >90% of the positions, the resulting alignment was used to construct a phylogenetic tree with FastTree (Price et al. 2009).

For further analysis, the bacterial communities (full OTU tables) were normalized to the minimum sequencing depth (i.e. 5557 sequences) associated with 100 bootstrap resampling runs. The number of observed OTUs (OTU richness) was calculated using the vegan package (Oksanen et al. 2018) in R v. 3.5.0 (R Core Team 2018). The relationship between OTU richness (based on an average of 100 bootstraps) and distance to the uppermost end (i.e. Station HM01) was tested using linear regression or generalized additive model in R. The rarefied bacterial communities were also used to generate the weighted UniFrac dissimilarity, which was further plotted using principal coordinate analysis (PCoA) using the vegan package.

### Quantitative estimate of assembly processes

To quantitatively examine dispersal- and selection-inferred ecological processes, we used the null model approach (Stegen et al. 2016) separately for the four sets (total and active; summer and winter) of bacterial communities. First, we tested the phylogenetic signals using Mantel correlograms to determine the relationship between phylogenetic distance and niche differences (inferred from all environmental variables) among OTUs prior to conducting null model analyses (Stegen et al. 2013, Dini-Andreote et al. 2015). Only OTUs (in the full OTU tables) with an occupancy (i.e. number of sites occupied) of >3 were included when testing the phylogenetic signals because an extremely low occupancy might bias the detection of niche specialization. Then, the phylogenetic turnover in the (rarefied) bacterial communities was quantified based on β-mean nearest taxon distance (βMNTD) using the picante package (Kembel et al. 2010). To infer the relative importance of different ecological processes, we calculated the pairwise β-nearest taxon index (βNTI), which accounted for the difference between the observed βMNTD and the mean of the null distribution normalized by its standard deviation (999 randomizations). βNTI values of ≤−2 and ≥+2 indicate significantly less than and more than the expected phylogenetic turnover, respectively (i.e. homogeneous and heterogeneous selection, respectively). In cases where |βNTI| < 2, we calculated the Raup–Crick metric based on Bray–Curtis dissimilarities (RC_bray_) (Stegen et al. 2013). Pairwise RC_bray_ values of >+0.95, <−0.95, and |RC_bray_| < 0.95 accounted for the relative contributions of dispersal limitation, homogenizing dispersal, and the undominated fraction (mainly indicating drift under weak dispersal- and selection-inferred processes), respectively (Dini-Andreote et al. 2015, Stegen et al. 2015, Zhou and Ning 2017). The bootstrap analysis for 100 replicates was included in the null model analyses.

As described above, when determining a driving ecological process for pairwise bacterial communities, the number of OTUs (corresponding to the nodes of the phylogenetic tree used for the calculation of βMNTD) and their relative abundances are important. This is because these two community characteristics can essentially affect the values of βNTI and RC_bray_ based on 999 randomizations. To test the effect of OTU numbers and relative abundances, we further calculated ecological processes shaping the sub-communities composed of the most abundant 1000 OTUs. The resulting difference in ecological processes (compared to those of whole bacterial communities) supports the effect of diversity (i.e. OTU numbers) and rarity (i.e. relative abundances of OTUs) on null model analyses.

### Habitat preference of OTUs

To identify OTUs that are specifically associated with different habitats (i.e. freshwater, oligohaline, mesohaline, polyhaline, and marine environments), the analysis of indicator value (IndVal) was performed based on relative abundances (in the full OTU tables) using the indicspecies package (De Cáceres and Legendre 2009). The OTUs with a preference of a particular habitat were recognized as having significant (*P* < 0.05) IndVal estimations (ranging from 0 to 1) through 999 permutations (Hauptmann et al. 2016). OTUs showing a relative abundance of <0.01% in all the samples were removed before IndVal analyses because their rarities biased the prediction of habitat preference.

### Statistical analysis

To visualize community characteristics, rank–abundance and rarefaction curves were plotted using the vegan package with the radfit and rarefy functions, respectively. Moreover, the importance of each environmental factor in promoting beta diversity (weighted UniFrac dissimilarity) was individually tested using permutational multivariate analysis of variance (PERMANOVA, 999 permutations) with the adonis2 function in the vegan package. The PERMANOVA was also used to examine the difference between the total and active bacterial communities (999 permutations). To assess the community assembly processes along the PSC, we regressed pairwise βNTI values (using an average of 100 bootstraps) against environmental distances (Kumari et al. 2015, Stegen et al. 2016, Feng et al. 2018, Zhang et al. 2018).

## Results

### Hydrographic condition

There were a total of 49 and 37 significant correlation relationships of the 13 environmental variables in summer and winter, respectively (Supplementary Fig. S1). As a typical hydrographic context in this river–sea continuum, salinity showed significantly negative correlations with nutrients (NH_4_, NO_2_, NO_3_, and PO_4_), particulate organic carbon, and particulate nitrogen in both seasons. Environmental distances among sites significantly increased with geographical distances based on Mantel tests (summer: *r* = 0.77, *P* < 0.001; winter: *r* = 0.76, *P* < 0.001). Moreover, a total of 6 and 0 samples located in the freshwater habitat in summer and winter, respectively (Fig. 1B), indicating that this transect was more influenced by freshwater in the wet season due to more river runoffs.

### Community characteristic

The sum of OTUs detected in summer and winter were 45 594 and 40 328, respectively, a considerable percentage of which was shared (summer, 43%; winter, 49%) between total and active bacterial communities (Supplementary Fig. S2A and B). The rank–abundance curves displayed a long tail (Supplementary Fig. S2C and D), and the rarefaction curves of individual bacterial communities generally did not reach an asymptote (Supplementary Fig. S2E).

Total bacterial communities showed significantly (*P* < 0.05) higher OTU richness than their active counterparts (Supplementary Fig. S3). Moreover, OTUs in total bacterial communities had higher habitat occupancies than those in active fractions, when compared based on a given relative abundance (Supplementary Fig. S4). Remarkably, the most abundant 10 OTUs in each dataset, except an OTU in active bacterial communities in summer (OTU49), were detected in all samples (Supplementary Fig. S5).

**Figure 2.**
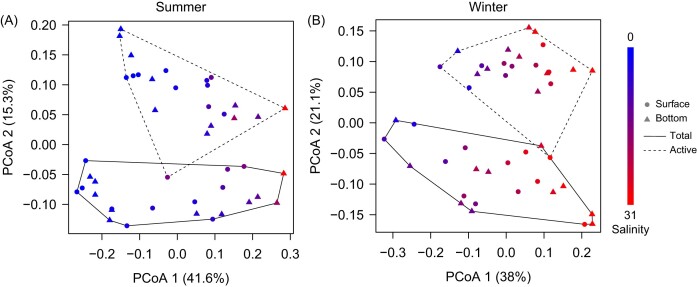
PCoA plots based on weighted UniFrac dissimilarities during (A) summer and (B) winter associated with changes in salinity, which are indicated by colors such that blue is 0 and red is 31. The total and putatively active bacterial communities found in the surface and bottom samples collected at each season are indicated by different lines and symbols, respectively. Each point represents the average derived from 100 bootstraps. The only environmental factor that was significantly (*P* < 0.05) related to community dissimilarities in all four sets of bacterial communities was salinity (Supplementary Table S1). The total and active bacterial communities were shown to be significantly different using PERMANOVA (summer, *R*^2^ = 0.14, *P* < 0.001; winter, *R*^2^ = 0.2, *P* < 0.001).

The PCoA ordination revealed that total and active bacterial communities were significantly different (*P* < 0.001), both of which shifted along salinity gradients (Fig. 2). Furthermore, salinity was the only environmental factor that was significantly (*P* < 0.05) related to community dissimilarities in all four sets of bacterial communities (Supplementary Table S1).

### Community assembly

**Figure 3.**
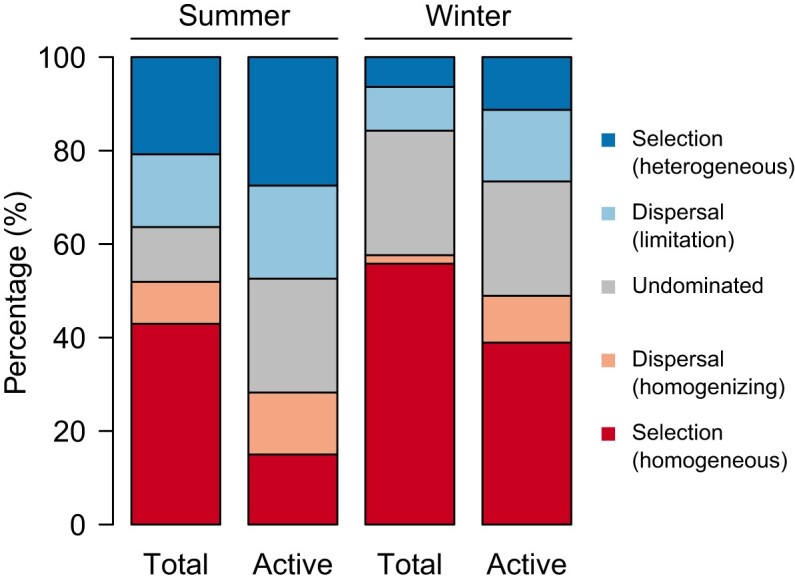
Barplots showing the proportions of ecological processes (based on 100 bootstraps) in shaping total and active bacterial communities in summer and winter seasons.

The null model analyses, supported by the phylogenetic signal particularly pronounced among OTUs with short phylogenetic distances (Supplementary Fig. S6), showed that ecological processes on average comprised 38.2% homogeneous selection, 8.5% homogenizing dispersal, 21.8% undominated fraction (indicating weak dispersal- and selection-inferred processes), 15% dispersal limitation, and 16.5% heterogeneous selection (Fig. 3). In detail, heterogeneous selection exceeded homogenizing dispersal in all four sets of bacterial communities, in spite of a slightly different proportion in the active bacterial communities in winter (heterogeneous selection, 11.3%; homogenizing dispersal, 10%).

**Figure 4.**
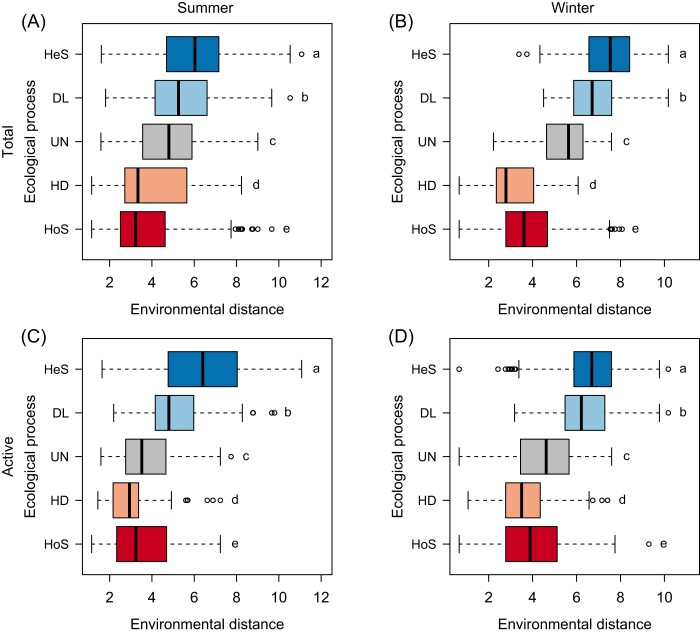
Boxplots showing the environmental distances in which each ecological process was observed in the (A, B) total and (C, D) active bacterial communities. The boxplots display five summary statistics, including median (black line), 25% and 75% percentiles (box), and range (whiskers), and outliers are indicated by circles. The different letters (a–e) next to each boxplot indicate the significant differences in pairwise comparisons based on the Kruskal–Wallis rank sum test (*P* < 0.001). The ecological process showing a mean in the middle within the groups of environmental distances, are simply designated as “occurring at moderate environmental distances.” Accordingly, ecological processes showing a higher or lower mean are defined as “occurring at high or low environmental distances,” respectively. HeS, heterogeneous selection; DL, dispersal limitation; UN, undominated fraction; HD, homogenizing dispersal; and HoS, homogeneous selection.

In either summer or winter seasons, homogeneous and heterogeneous selection showed higher proportions in total and active bacterial communities, respectively (Fig. 3). Notably, ecological processes shifted with changes in environmental distance (Fig. 4), characterized by significant linear regression relationships (*P* < 0.001) between environmental distances and βNTI values (Supplementary Fig. S7). βMNTD also increased with increasing environmental distances (Supplementary Fig. S8).

The assembly of the sub-communities of the most abundant 1000 OTUs was on average comprised of 0.04% homogeneous selection, 9.8% homogenizing dispersal, 23.8% undominated fraction, 21.7% dispersal limitation, and 44.6% heterogeneous selection (Supplementary Fig. S9). Moreover, heterogeneous selection was the highest except in the total bacterial communities in winter showing an undominated fraction of 39.3% (heterogeneous selection = 31.5%).

**Figure 5.**
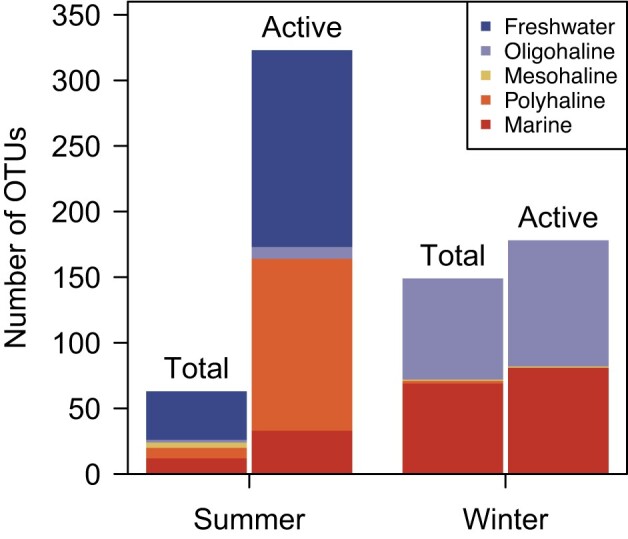
Barplots showing the numbers of OTUs with habitat preference in the five types of habitats (freshwater, oligohaline, mesohaline, polyhaline, and marine).

In addition, accompanied with the difference in ecological processes between total and active bacterial communities (Fig. 3), the former showed less OTUs with habitat preference than the latter in both summer (total, 63; active, 323) and winter (total, 149; active, 178) (Fig. 5).

## Discussion

Our study found that heterogeneous selection exceeded homogenizing dispersal in shaping bacterial communities in the PSC, implying that environmental gradients are more important than geographic connections. In each season, homogeneous and heterogeneous selection showed higher contributions in total and active bacterial communities, respectively, supporting that the metabolic activity makes a difference in bacterial community assembly.

### Ecological process shifting in the PSC

Ecological processes shaping bacterial communities mainly occurred under different environmental distances in the PSC (Fig. 4 and Supplementary Fig. S7). We divide the environmental distances into three levels for ease of interpretation: small (mainly showing homogeneous selection and homogenizing dispersal), moderate (mainly showing undominated fraction), and large (mainly showing dispersal limitation and heterogeneous selection). Specifically, under small environmental distances (accompanied by small geographical distances), the passive dispersal of bacteria can be efficiently facilitated (Heino et al. 2015). However, homogenizing dispersal has to compete against homogeneous selection in this scenario (Souffreau et al. 2014). Homogeneous selection was prevalent (Fig. 3) based on estimations in the first step of the null model analysis, which therefore weakens the role of homogenizing dispersal determined in the second step.

In contrast, the large environmental distances act as strong selective forces for bacterial taxa with extremely distinct evolutionary histories showing scarce freshwater-marine transitions (Logares et al. 2009, Newton et al. 2011, Herlemann et al. 2016, Paver et al. 2018). For example, selective pressures such as salinity-related mortality (Painchaud et al. 1995) can efficiently differentiate bacterial communities (Fig. 2). Moreover, distinct trends arose at the OTU level in freshwater- and seawater-like habitats with relative abundances of the most abundant OTUs peaking at different locations across the transect (Supplementary Fig. S5). These differential patterns under large environmental distances can be partly attributable to heterogeneous selection primarily responsible for the large phylogenetic turnover between bacterial communities (Supplementary  Fig. S8). Alternatively, dispersal limitation emerged (Fig. 4 and Supplementary Fig. S7) because the majority of bacterial individuals are unable to disperse across strong environmental gradients. Therefore, our results support that dispersal limitation can arise not only from geographical barriers but also through interactions with environmental heterogeneities (Adler et al. 2007).

The undominated fraction was common among sites with moderate environmental distances (Fig. 4 and Supplementary Fig. S7). On the one hand, sites with moderate environmental distances are linked via water flow, which can greatly reduce dispersal limitation but may not be strong enough to generate homogenizing dispersal. On the other hand, selective forces such as changing salinity (Lozupone and Knight 2007) under moderate environmental distances can largely reduce homogeneous selection, but may not be strong enough to generate heterogeneous selection. Most bacteria from freshwater are stressed when transported to downstream stations, and may appear to be randomly filtered in rapidly changing environments (i.e. by drift). Therefore, the occurrence of undominated fraction is responsible for the scenario in which no single process inferred from either dispersal or selection is dominant (Zhou and Ning 2017).

In our bootstrapped results (*n* = 100), the assembly of the same community pair (i.e. dots sharing an x-axis, Supplementary  Fig. S7) could be attributed to different ecological processes (i.e. in different colors). To some degree, this is consistent with the idea of reconciling dispersal- and selection-based perspectives in metacommunity ecology (Gravel et al. 2006); that is, dispersal and selection can be jointly responsible for differences between each pair of bacterial communities, despite only one ecological process recognized in null model analyses. Alternatively, rarefaction is a random process that can cause instabilities in null model analyses (Ma and Tu 2022). That is, rarefying the largest sample of 59 963 sequences into 5557 sequences (i.e. minimum sequencing depth) may result in some compositional instability among the subsampled communities, influencing the ecological processes in the subsequent analyses. Nonetheless, we suggest that including a bootstrap step with repeatedly subsampling (Schloss 2024) can get more accurate quantifications of ecological processes.

### Prevalence of homogeneous selection

Our results showed that homogeneous selection can emerge in ecosystems with strong environmental gradients, consistent with several recent studies with similar observations (Wang et al. 2020, Wu et al. 2021, Urvoy et al. 2022, Blais et al. 2024). Moreover, our study expands the prevalence of homogeneous selection that has been observed in the oligotrophic South Pacific Gyre (Allen et al. 2020), hydrologically connected rivers (Graham et al. 2017, Cai et al. 2019), glacier-fed streams (Fodelianakis et al. 2022), and proglacial floodplain streams (Brandani et al. 2023).

A few characteristics related to bacterial communities can primarily enhance the strength of homogeneous selection. First, the bacterial communities in the PSC are tremendously diverse in part because they received allochthonous inputs of microorganisms from soil and sediments, as well as a mix of freshwater and marine taxa (Crump et al. 2012). The PSC had a large number of OTUs during summer (total, 41 582; active, 23 611) and winter (total, 34 309; active, 25 790). Meanwhile, both total and active bacterial communities exhibited a high degree of rarity (indicated by the long tails in the rank–abundance curves, Supplementary  Fig. S2C and D), which is a common property in bacterial communities (Pedrós-Alió 2012). Second, sites with small environmental distances may share some OTUs with high relative abundances (for convenience, simply designated as abundant OTUs). Taking the most abundant 10 OTUs for example, they commonly showed comparable relative abundances between neighboring sites (Supplementary  Fig. S5). As a result, the phylogenetic distances between two bacterial communities (quantified by the observed βMNTD) under small environmental distances were relatively low (Supplementary  Fig. S8) because this metric is determined by the phylogenetic positions and relative abundances of OTUs. More importantly, the null expectation for pairwise community distances is derived by randomizing OTUs across the phylogeny and recalculating βMNTD 999 times (Stegen et al. 2012), and thus the observed βMNTD values may be much lower than the null expectation based on tremendous diversity associated with high rarity. A resulting high degree of deviation of βMNTD from the null expectation (i.e. βNTI ≤ −2) is a sign of homogeneous selection.

When focusing on sub-communities composed of the most abundant 1000 OTUs (i.e. lowering the diversity and rarity), we found that homogeneous selection was significantly negated with contributions approximately equal to 0 (Supplementary  Fig. S9). Similar to our examination regarding diversity associated with rarity, a recent study conducted in inshore waters showed the dominance of non-selection processes in shaping bacterial sub-communities of the most abundant 500 OTUs (Han et al. 2022). This is because both diversity and rarity are intensively lowered in null modeling, leading to a low degree of deviation of βMNTD from the null expectation (i.e. |βNTI| < 2). Another recent study showed that the relative importance of homogeneous selection increases with increasing similarity cutoffs for grouping OTUs (Quiroga et al. 2022). This is primarily due to the fact that using a higher similarity cutoff (i.e. a higher phylogenetic resolution) can largely increase both diversity (i.e. having an expanded phylogenetic tree when calculating βMNTD) and rarity (i.e. lowering the sequence number per OTU) for a given dataset. In addition, the sharing of abundant OTUs between sites (as mentioned above) benefits from our gradient-type sampling design (including freshwater, oligohaline, mesohaline, polyhaline, and marine habitats). Given a coarser sampling, e.g. by only sampling the two ends of the PSC (i.e. HM01 and ZJ09), the selection would be mainly heterogeneous rather than homogeneous (Supplementary  Fig. S7). This is consistent with the notion that community assembly is scale-dependent (Chase 2014, Ladau and Eloe-Fadrosh 2019, Langenheder and Lindström 2019). Therefore, we suggest that the prevalence of homogeneous selection in this study is primarily attributable to the bacterial communities’ tremendous diversity (associated with high rarity) and our specific sampling design.

### Total versus active bacterial communities

As expected, homogeneous and heterogeneous selection in each season were more important in total and active bacterial communities, respectively (Fig. 3), consistent with that active bacteria are more responsive to environmental gradients than are the total bacteria (De Vrieze et al. 2016). This is supported by the observation that active bacterial communities had more OTUs showing habitat preferences than total bacterial communities (Fig. 5). Moreover, OTUs in the total communities exhibited consistently higher occupancies than those in the active fractions (Supplementary  Fig. S4), also implying that the former could be less sensitive to environmental gradients by inhabiting a wider variety of niches. Notably, the higher occupancies of OTUs in total bacterial communities may primarily result from the inclusion of dead members, because dead bacteria can potentially persist in habitats ranging from freshwater to marine. This interpretation is in line with a study demonstrating that marine bacteria can even be recruited from freshwater sources in DNA-inferred communities (Comte et al. 2014).

However, when interpreting the differences in community assembly, it should be noted that active bacterial communities are not a simple subset of total communities. For example, a significant proportion of OTUs was found only in the active communities (summer = 8.8%, winter = 14.9%) (Supplementary  Fig. S2A and B), and seven active communities had higher OTU richness than their total counterparts (Supplementary  Fig. S3). The OTUs found exclusively in active communities could be extremely rare taxa from the rare biosphere (Sogin et al. 2006). The detection limitation in sequencing methodology contributes to this discrepancy because DNA-based data might lose some rare taxa that were solely recovered in RNA-based data (and *vice versa*). Alternatively, these OTUs could have been derived from RNA that survives outside the organismal environment (Cristescu 2019). Moreover, examining the assembly of either total or active bacterial communities is complicated and influenced by a variety of factors. For example, it is well known that both rDNA (Stoddard et al. 2015) and rRNA (Kirchman 2016) copy numbers vary greatly among bacterial taxa, with rRNA copy numbers in particular varying by several orders of magnitude for marine bacterial strains at various metabolic stages (Fegatella et al. 1998). As a result, when comparing the assembly of total and active bacterial communities, their skewed community structures are a significant source of biased interpretations (Lavrinienko et al. 2021).

## Conclusions

**Figure 6.**
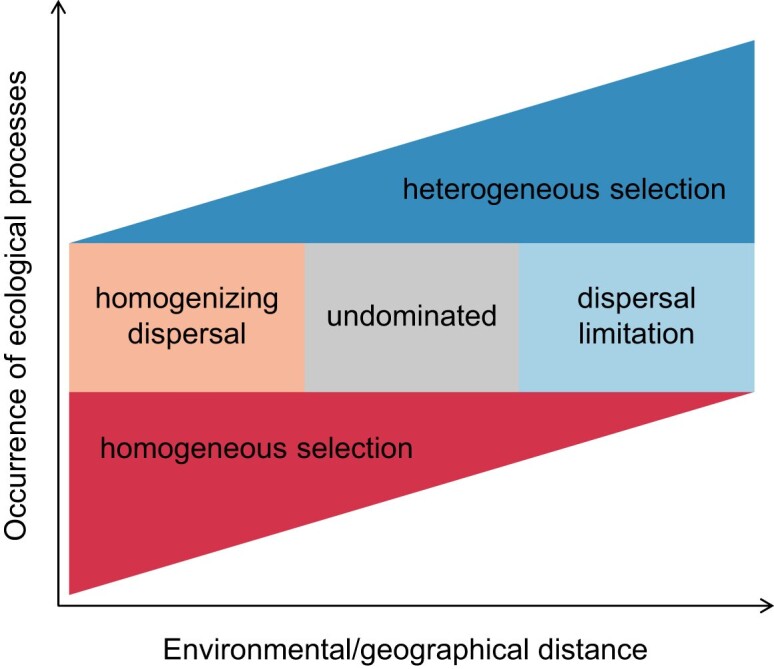
A conceptual model indicates the environmental dependency of ecological progresses in a river–sea continuum. In the event of low environmental distances (commonly showing significant correlations to geographical distances), homogeneous selection and homogenizing dispersal occur. Under moderate environmental distances, neither dispersal- nor selection-inferred processes are dominant and so the fraction is undominated. Under large environmental distances, dispersal limitation and heterogeneous selection become the major processes. This progressive succession in ecological processes could result in a significant linear regression relationship between βNTI values and environmental distances.

Our study makes inferences about how highly connected bacterial communities are assembled along strong environmental gradients (Fig. 6). Specifically, homogeneous selection and homogenizing dispersal occur primarily in small environmental distances; the undominated fraction mainly emerges in moderate environmental distances and mostly indicates drift; dispersal limitation and heterogeneous selection occur primarily in large environmental distances. In the PSC, heterogeneous selection exceeds homogenizing dispersal in all four sets of bacterial communities, and homogeneous and heterogeneous selection (in each season) are more important in total and putatively active bacterial communities, respectively. Moreover, our study reveals that calculating ecological processes can be heavily influenced by a few community characteristics (e.g. diversity and rarity). This suggests that when interpreting the ecological processes, the phylogenetic and proportional properties of a metacommunity should be carefully considered. In summary, this study emphasizes the importance of disentangling community assembly mechanisms to understand microbial biogeography in natural ecosystems.

## Supplementary Material

fiae146_Supplemental_File

## Data Availability

Raw sequencing data are available from the NCBI Sequence Read Archive under PRJNA805376.
