# Supplementary material for: Ecological processes shaping highly connected bacterial communities along strong environmental gradients
Source: FEMS Microbiol Ecol. 2024 Oct 30;100(12):fiae146. doi: 10.1093/femsec/fiae146 (PMC11687216; doi:10.1093/femsec/fiae146)

**Supporting information**

**Table S1.** Environmental factors (within all 13 parameters) showing significant relationships (*P* < 0.05) with total and putatively active bacterial community dissimilarities supported by PERMANOVA (999 permutations). In general, the *R*^2^ values are shown but data that are not available due to non-significant relationships are indicated by ‘–’.

|  | Summer, total | Summer, active | Winter, total | Winter, active |
| --- | --- | --- | --- | --- |
| Temperature | 0.37 | 0.24 | – | – |
| Salinity | 0.33 | 0.22 | 0.57 | 0.39 |
| NO_2_ | 0.07 | 0.08 | – | – |
| *Synechococcus* | – | – | 0.1 | 0.11 |

**Figure S1.** Heatmap showing significant (*P* < 0.05) Pearson correlation coefficients (circles) when comparing environmental variables during (A) summer and (B) winter. Positive and negative correlations are shown in blue and red, respectively. T, temperature; S, salinity; Chl, chlorophyll *a*; SPM, suspended particulate matter; POC, particulate organic carbon; PN, particulate nitrogen; Peuk, pigmented picoeukaryotes; Syne, *Synechococcus*; and HB, heterotrophic bacteria.


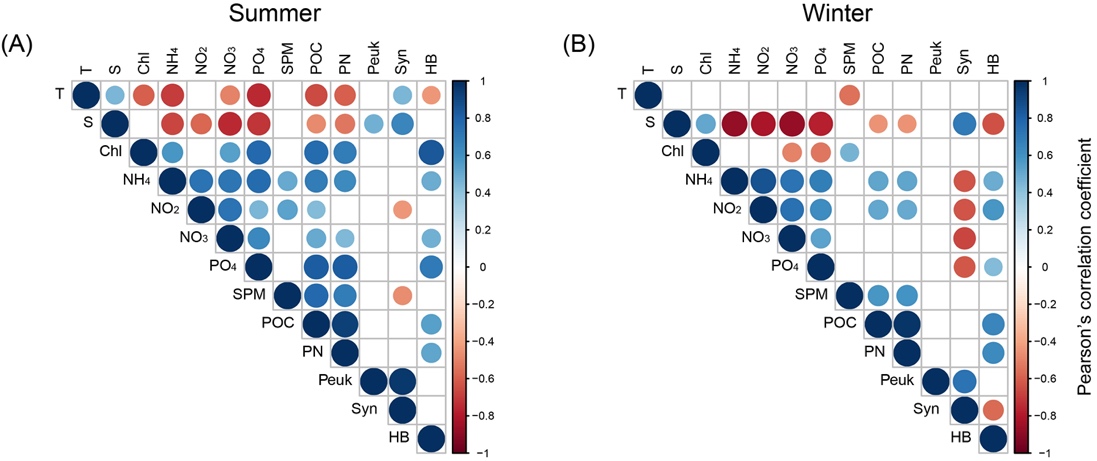


**Figure S2.** Venn diagrams (A, summer; B, winter) of observed OTU numbers and rank–abundance curves (C, summer; D, winter) for the total and active bacterial communities. Rarefaction curves of individual samples are provided (E). In panels (C) and (D), the total and active communities are fitted using the Zipf and Lognormal models, respectively, which were chosen based on the lowest AIC (Akaike’s information criterion) values; and the long tails indicate a high degree of rarity. All analyses are based on the full OTU tables.


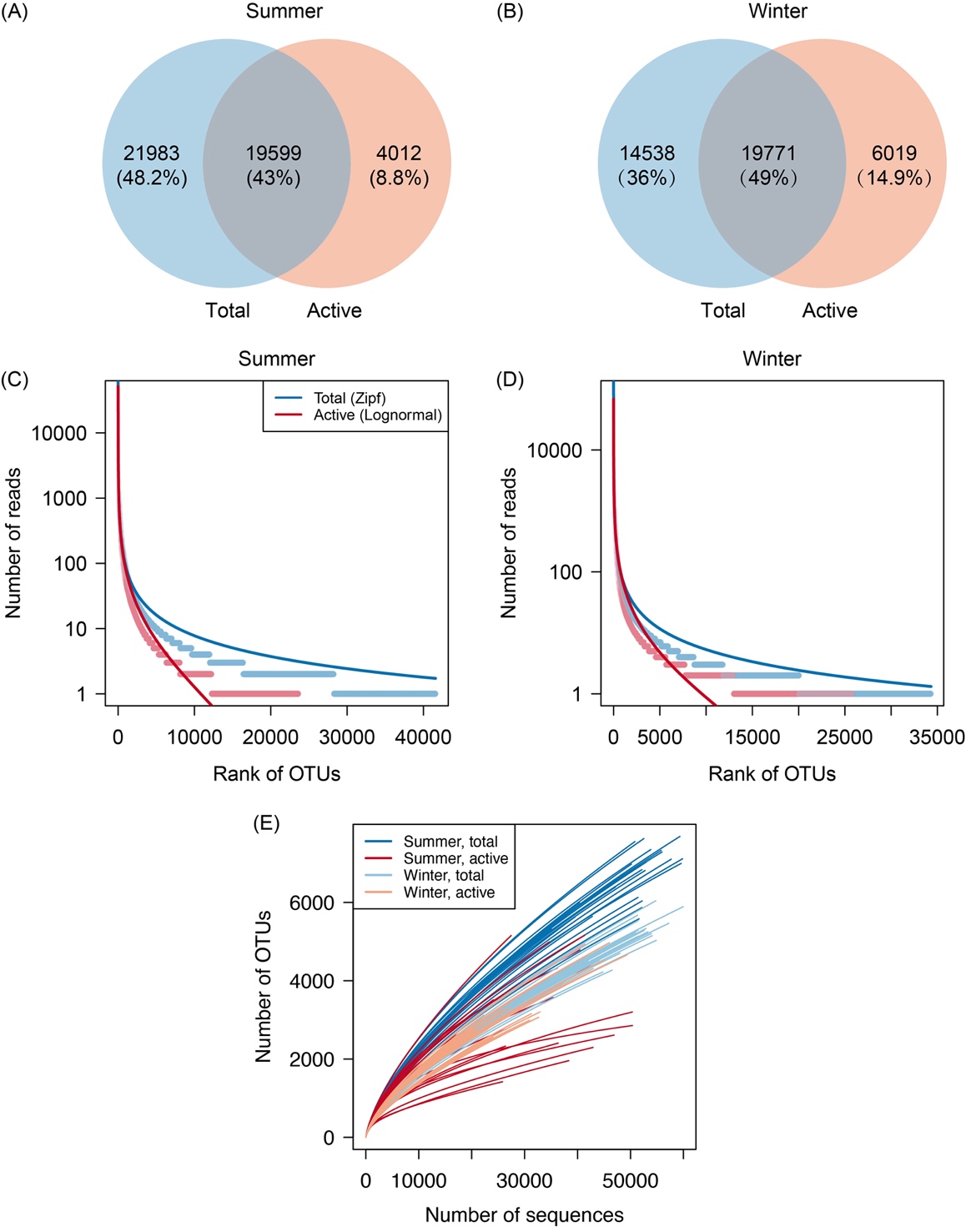


**Figure S3.** Plots illustrating the OTU richness within each community rarified to 5,557 reads (A, summer; B, winter). Linear regression was performed for the total bacterial communities, and generalized additive models (GAMs) were used to explore patterns in the active communities. The solid lines represent the linear (total) or GAM smooth (active) trends, and the dashed lines indicate the 95% confidence intervals. Each point represents the average derived from 100 bootstraps. Moreover, boxplots are added to summarize the OTU richness for the total and active bacterial communities, and the asterisks indicate the level of significance when the data were compared using the *t*-test, such that ** *P* < 0.001 and * *P* < 0.05.


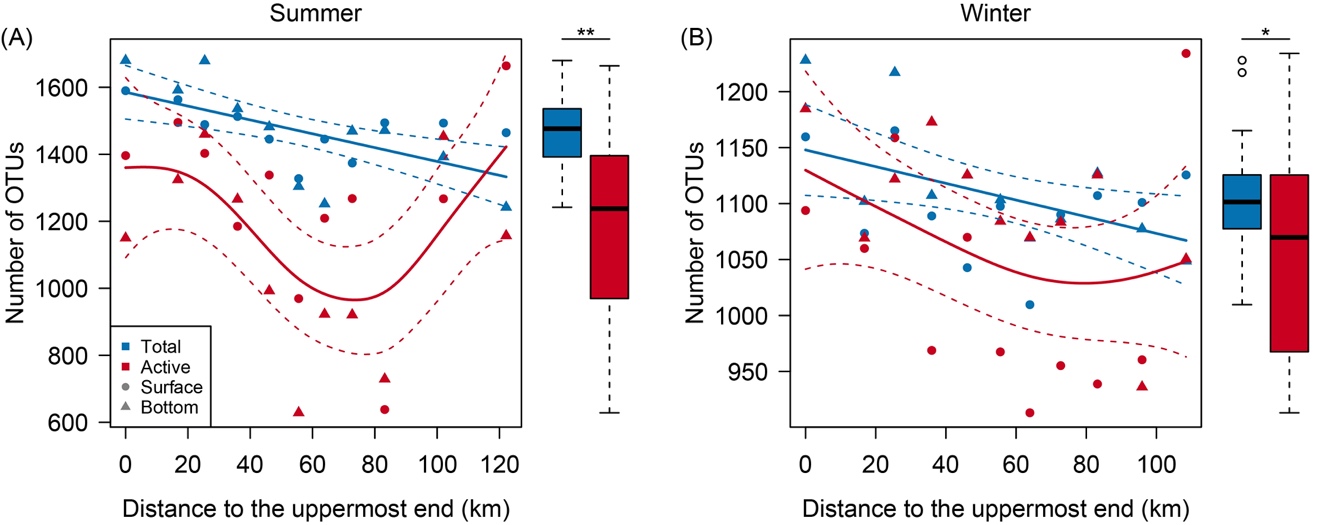


**Figure S4.** Abundance–occupancy relationship of the total and active OTUs during (A) summer and (B) winter. The mean relative abundances for OTUs were based on the full OTU tables (i.e., without rarefactions). The cubic smoothing spline is fitted to the abundance–occupancy relationships.

**
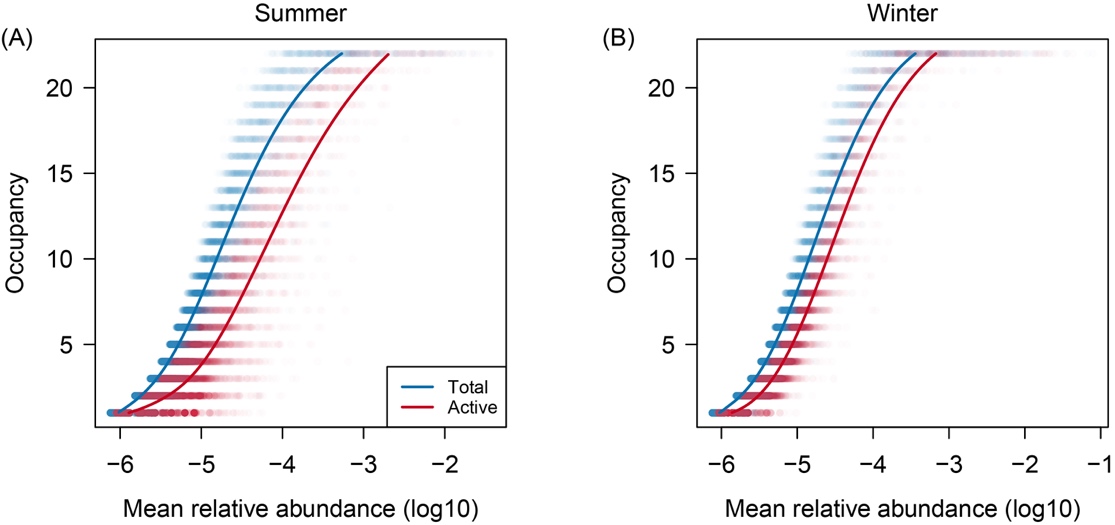
**

**Figure S5.** Spatial patterns of the 10 regionally most abundant OTUs in the (A, B) total and (C, D) active bacterial communities along the PSC during summer and winter. The mean values of relative abundances of OTUs across the transect are given in brackets below OTU names. The relative abundance level of each OTU was normalized to their maximum, and the tick marks along the y-axis indicate 0, 50, and 100%.


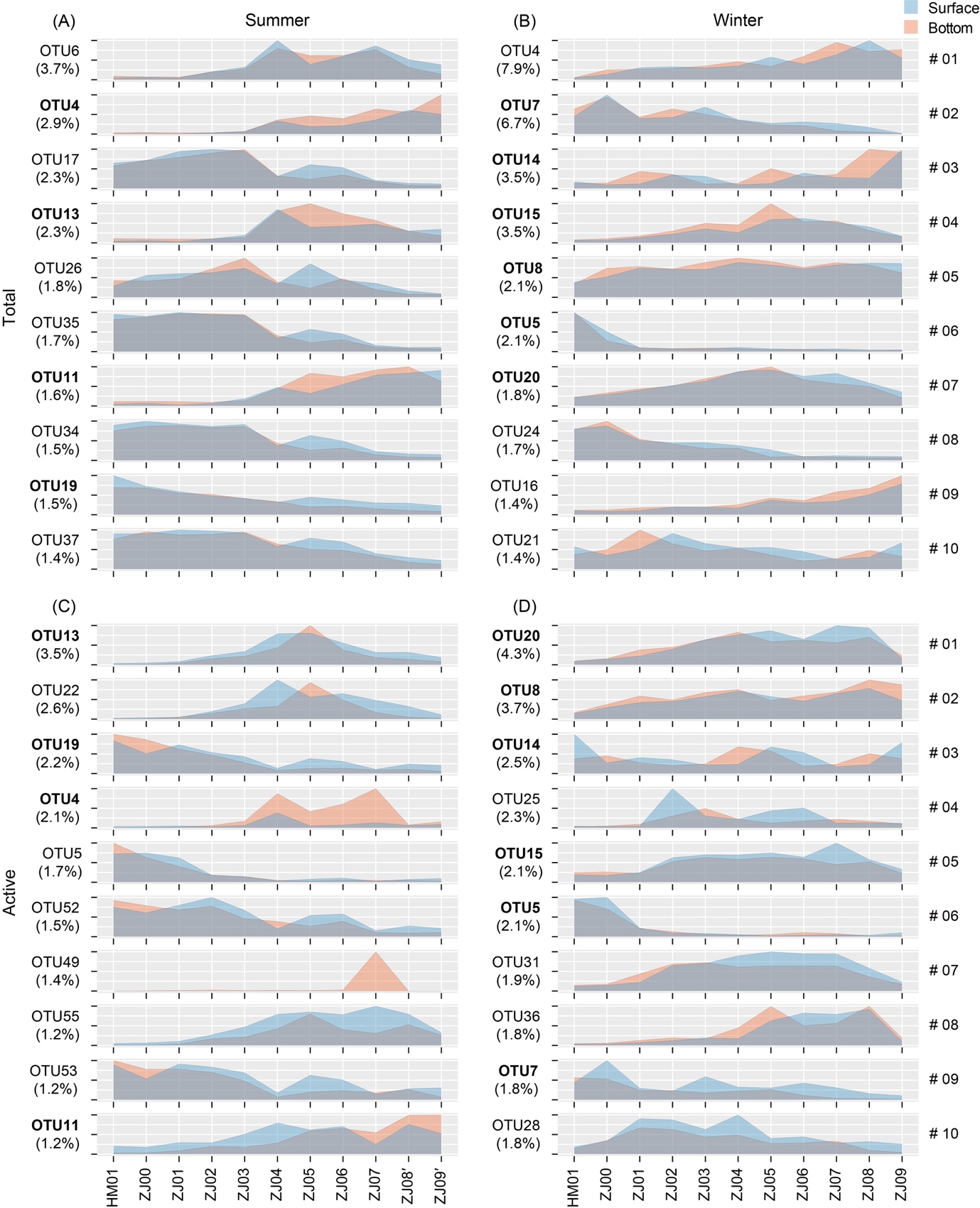


**Figure S6.** Pearson’s correlation coefficient (resulting from Mantel correlograms) of the pairwise matrix between environmental optima (indicating niche) and the phylogenetic distance of OTUs for the total (A, B) and active (C, D) bacterial communities collected during the summer and winter. The phylogenetic distance was standardized and ranged from 0 to 1 (50 phylogenetic distance classes). Significant correlations (*P* < 0.05, solid symbols) indicate phylogenetic signals of niches of OTUs, and non-significant correlations are indicated by open symbols. Phylogenetic signals were found especially among OTUs with short phylogenetic distances.

**
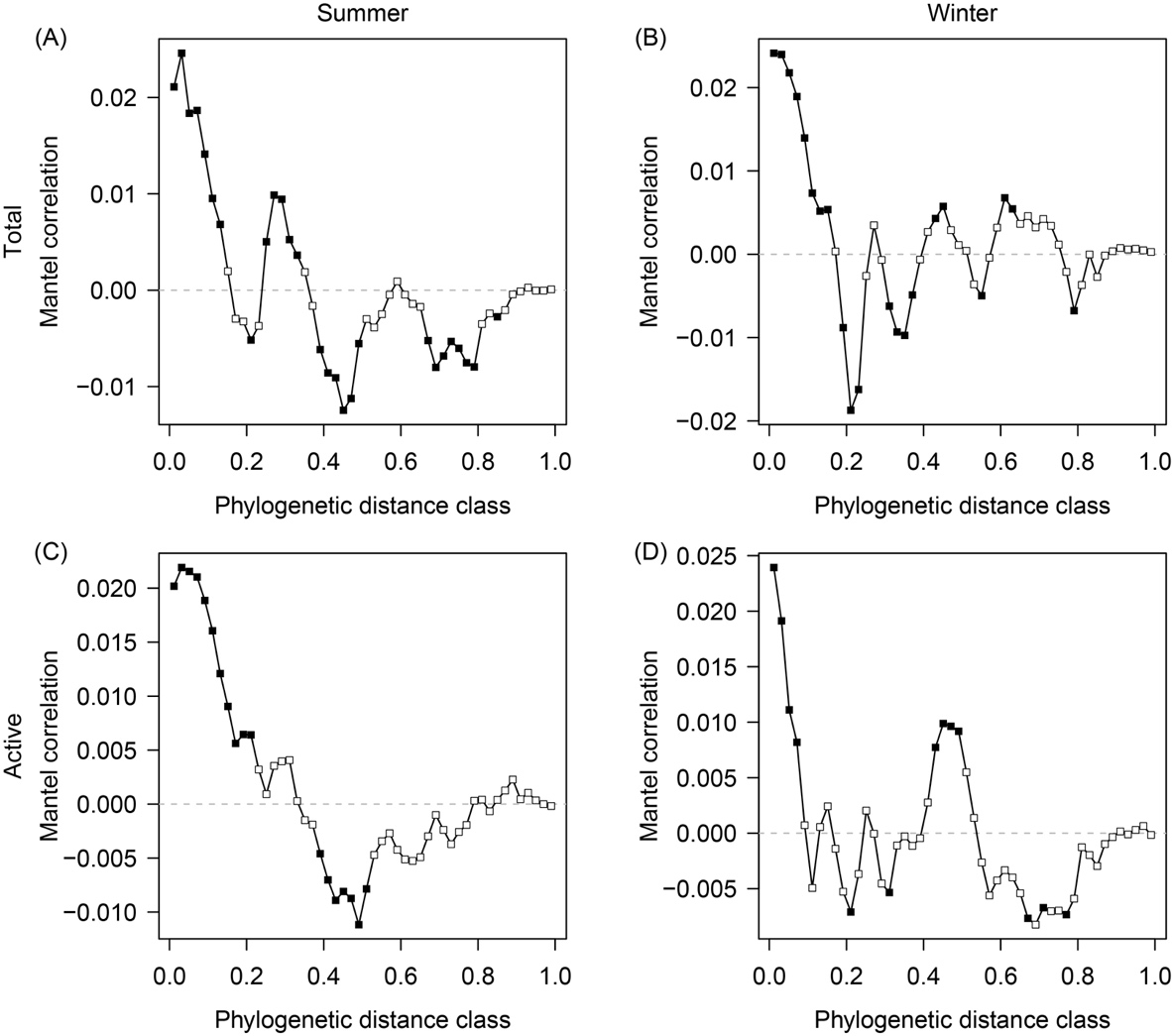
**

**Figure S7.** Relationship between βNTI and environmental distance for (A, B) total and (C, D) putatively active bacterial communities across the PSC. The βNTI values were calculated using the null model framework, and the environmental distances were based on Euclidean distances of environmental variables between stations. The colored points represent observations (based on 100 bootstraps) that are divided into five categories indicating different community assembly processes. The solid lines indicate significant linear regression relationships (*P* < 0.001), and the dashed lines indicate βNTI values of –2 or +2. The proportions of color dots (i.e., percentages of ecological processes) are shown in Fig. 3, while the environmental distances where ecological processes occur are summarized in Fig. 4.

**
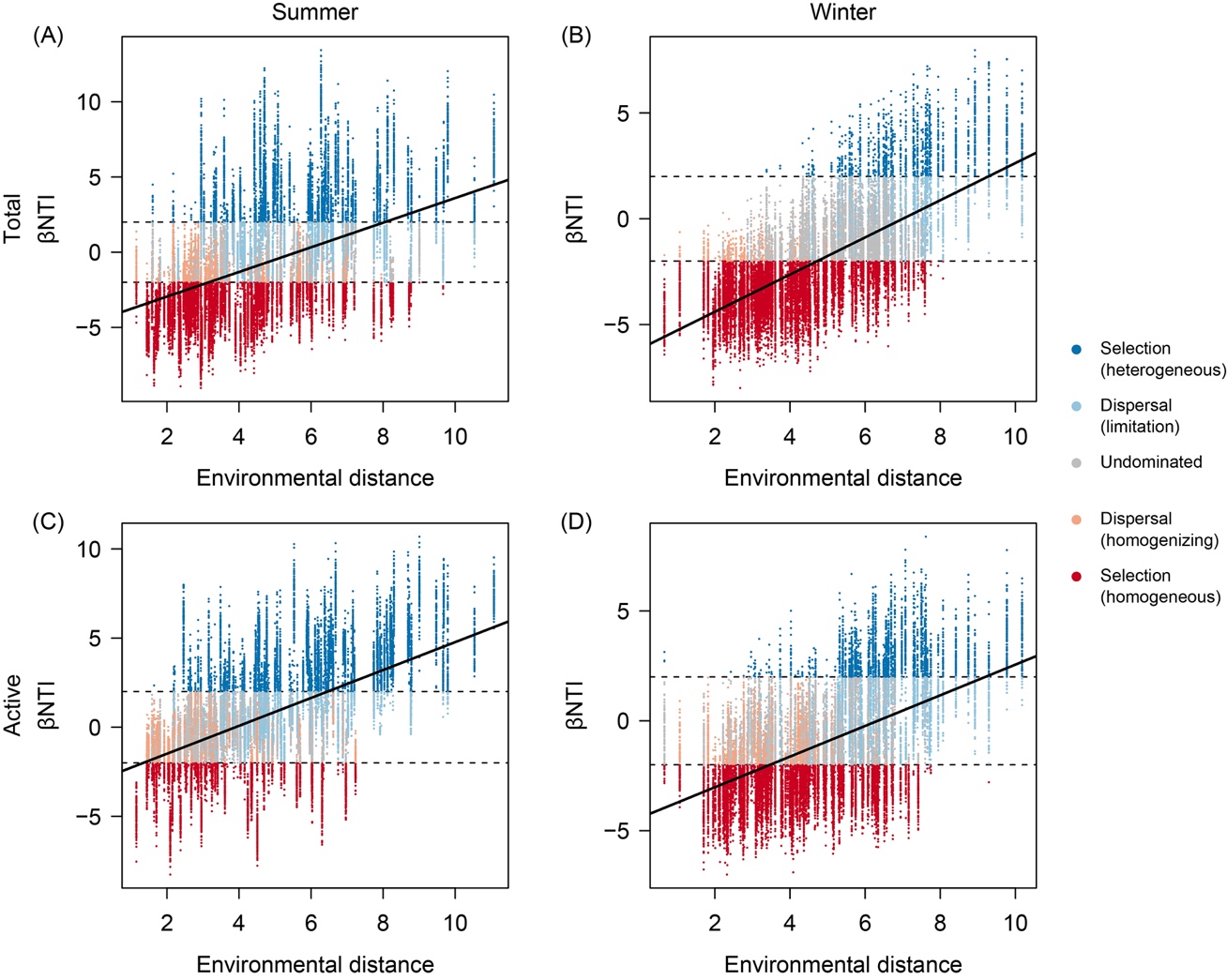
**

**Figure S8.** Relationship between environmental distance and observed βMNTD (average of 100 bootstraps) in the total and active bacterial communities in the summer (A) and winter (B). The cubic smoothing spline is fitted to the environmental distance–βMNTD relationships. In general, βMNTD values (i.e., phylogenetic turnover) increase when environmental distances increase.


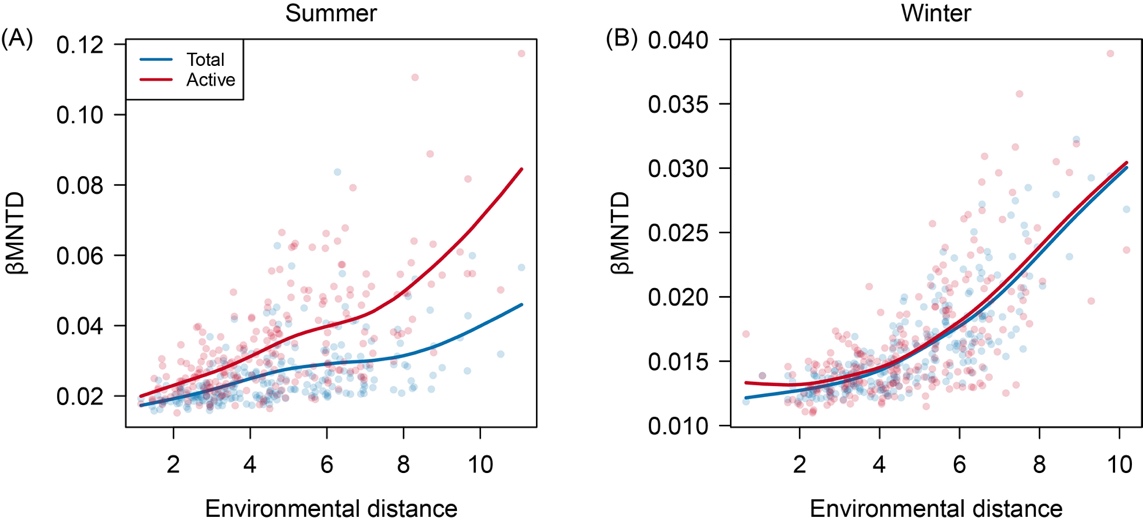


**Figure S9.** Barplots showing the proportions of ecological processes (based on 100 bootstraps) of the sub-communities comprised of the most abundant 1,000 OTUs. On average (taking all four sets together), ecological processes account for 0.04% homogeneous selection, 9.8% homogenizing dispersal, 23.8% undominated fraction (indicating weak dispersal- and selection-inferred processes), 21.7% dispersal limitation and 44.6% heterogeneous selection of the community variations.


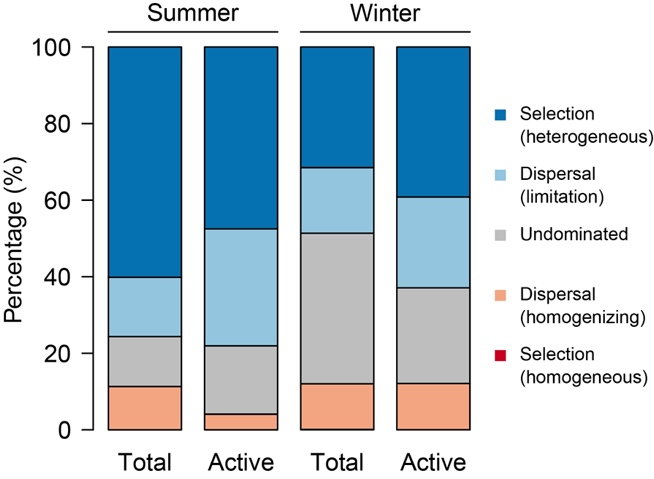

Supplement: fiae146_Supplemental_File [file fiae146_supplemental_file.docx]
